# Allografts for partial meniscus repair: an *in vitro* and *ex vivo* meniscus culture study

**DOI:** 10.3389/fbioe.2023.1268176

**Published:** 2023-10-12

**Authors:** Mohammad Dabaghi, Volker Eras, Daniel Kaltenhaeuser, Norus Ahmed, Britt Wildemann

**Affiliations:** ^1^ Experimental Trauma Surgery, Department of Trauma, Hand and Reconstructive Surgery, Jena University Hospital, Friedrich Schiller University Jena, Jena, Germany; ^2^ German Institute for Cell and Tissue Replacement (DIZG, gemeinnützige GmbH), Berlin, Germany

**Keywords:** meniscus lesion, biocompatible scaffold, allograft, collagen meniscus implant, knee

## Abstract

The purpose of this study was to evaluate the treatment potential of a human-derived demineralized scaffold, Spongioflex^®^ (SPX), in partial meniscal lesions by employing *in vitro* models. In the first step, the differentiation potential of human meniscal cells (MCs) was investigated. In the next step, the ability of SPX to accommodate and support the adherence and/or growth of MCs while maintaining their fibroblastic/chondrocytic properties was studied. Control scaffolds, including bovine collagen meniscus implant (CMI) and human meniscus allograft (M-Allo), were used for comparison purposes. In addition, the migration tendency of MCs from fresh donor meniscal tissue into SPX was investigated in an *ex vivo* model. The results showed that MCs cultured in osteogenic medium did not differentiate into osteogenic cells or form significant calcium phosphate deposits, although AP activity was relatively increased in these cells. Culturing cells on the scaffolds revealed increased viability on SPX compared to the other scaffold materials. Collagen I synthesis, assessed by ELISA, was similar in cells cultured in 2D and on SPX. MCs on micro-porous SPX (weight >0.5 g/cm^3^) exhibited increased osteogenic differentiation indicated by upregulated expression of ALP and RUNX2, while also showing upregulated expression of the chondrogen-specific SOX9 and ACAN genes. Ingrowth of cells on SPX was observed after 28 days of cultivation. Overall, the results suggest that SPX could be a promising biocompatible scaffold for meniscal regeneration.

## 1 Introduction

Human menisci are cartilaginous structures that provide cushioning to the knee joint by increasing the contact area of the tibiofemoral joint. The meniscus is composed of chondrocytes and fibroblasts that are embedded in an extracellular matrix composed of collagen, proteoglycans, glycoproteins and elastin (Chevrier et al., 2009; Makris et al., 2011). Meniscal lesions are a frequent occurrence in the workplace, during sports, and in daily activities. This may arise from the amalgamation of axial loading and rotational force, resulting in a shear load on the meniscus (Hede et al., 1990; Messner and Gao, 1998; Mather et al., 2015; Cao and Chen, 2022). The symptoms of meniscus lesions include pain, swelling, and difficulty moving the knee joint. In certain instances, the tear may result in the knee locking or catching when bent (Cao and Chen, 2022). A diagnosis is typically made through a physical exam and imaging, such as X-rays or MRI scans (Laible et al., 2013; Zhang, 2022).

Due to their poor vascularization particularly in their innermost regions, the menisci have a limited capacity for healing in the event of injury. In the past, a complete meniscectomy was performed as the standard of care. This would temporarily relieve symptoms, but in the long run, it would carry a higher risk of osteoarthritis (McDermott and Amis, 2006). Modern arthroscopic treatments typically focus on meniscal sutures or repairs, with a meniscectomy reserved for cases where such repairs are not possible. Some of the conventional treatment options for meniscus lesions include fixation devices, which are utilized to repair the meniscus tear and stabilize the joint (Brucker et al., 2010; Seo et al., 2020) and abrasion, which is a technique used to remove damaged tissue from the meniscus (Longo et al., 2012).

Over the past decades, regenerative medicine has come into focus to restore the specialized functions of the menisci. Several studies were conducted to develop and test materials for meniscus regeneration including hydrogels, polymer based fibers and scaffolds (Ronge et al., 2014; Vadodaria et al., 2019). The hydrogels can provide a supportive environment for the cells to regenerate (Romanazzo et al., 2018; Sasaki et al., 2018; Bansal et al., 2021), while the nanofibers can facilitate cell adhesion and proliferation (Hutmacher, 2001; Holloway et al., 2010; Holloway et al., 2014; Vadodaria et al., 2019). Additionally, the scaffolds provide a structure for the cells to attach to while allowing for the growth of new tissue (Whitehouse et al., 2017; Winkler et al., 2020; Angele et al., 2021; Hutchinson and Rodeo, 2022; Winkler et al., 2022). Each of these materials has the potential to improve the healing process and to restore the functionality of the meniscus. Only a few materials have been approved for human use. These include meniscal allografts (Cavendish et al., 2020) and cell-free biocompatible meniscus scaffolds (Scotti et al., 2013; Lombardo et al., 2021; Hutchinson and Rodeo, 2022).

Allograft transplantation is a procedure where healthy donor tissue is transplanted into the patient’s knee joint. The utilization of allogeneic materials for the treatment of meniscal lesions has been practiced for a considerable period of time (Kean et al., 2017). These materials are believed to serve as a scaffold for tissue regeneration and repair. However, the long-term outcomes of this technique, particularly in terms of chondroprotection and osteoarthritis prevention, have not yet been demonstrated (Bilgen et al., 2018). The potential disadvantages of this method include concerns about the availability of tissue, the immunological response, disease transmission, and the size of the graft (Scotti et al., 2013).

The use of a cell-free biomaterial for the restoration and replacement of a portion of the meniscus is supported by the potential for cell repopulation within the scaffold. The potential migration of cells from the synovium and meniscal residues, and the subsequent tissue integration, can lead to a post-implantation cell-based outcome (Scotti et al., 2013; Lu et al., 2022; Taghiyar et al., 2023). Veronesi et al. summarized the preclinical and clinical studies on the use of scaffolds for the treatment of partial meniscus lesions (Veronesi et al., 2021). Based on this review, only two scaffolds are approved for human use. The synthetic Actifit^®^ and the organic bovine collagen meniscus implant (CMI). As the initial regenerative technique, CMI was proposed as a treatment option for meniscal tissue regeneration in clinical practice (Stone et al., 1992; Zaffagnini et al., 2015). Initially, the clinical results of CMI implantation for partial meniscal regeneration were described as satisfactory (Buma et al., 2007). However, some studies have reported long-term shrinkage of the CMI, complete degradation, or the formation of scar tissue (Martinek et al., 2006; Scotti et al., 2013; Zaffagnini et al., 2015). A recent systematic review has revealed that the use of CMI as a singular effective treatment option for meniscal defects cannot be recommended for routine clinical application due to a lack of supporting evidence and a relatively high failure rate (Kohli et al., 2022).

The complexities and limitations of treating meniscal lesions, as well as the unsatisfactory outcomes of current procedures, necessitate the application of improved technologies and the use of biocompatible materials for innovative treatments. In this study, we investigated *in vitro* the suitability of a human-derived demineralized cancellous bone scaffold, called Spongioflex^®^ (SPX) for the treatment of partial meniscal lesions. Due to the different porosities of the native cancellous bone, SPX can be produced with different porosities. The SPX with a lower porosity has a higher weight (>0.5 g/cm^3^), while the SPX with higher porosity has a lower weight (<0.5 g/cm^3^). Demineralization of cancellous bone leads to elastic and flexible tissue properties. These properties give SPX a sponge like behavior which allows it to act as a shock absorber in addition to the scaffold properties. A recent case study on a patient with bilateral medial meniscal lesions revealed that SPX block implants resulted in rapid implant integration with good radiographic and functional outcomes after a short term follow up of 6 and 12 months respectively (right and left meniscus) (Behrendt, 2023). In the present study, we first investigated the differentiation of fresh meniscal cells (MCs) isolated from human donors in different culture and differentiation media. Secondly, we aimed to determine whether SPX provides a biocompatible scaffold that is capable of accommodating MCs for their regeneration while still preserving their fibroblastic and chondrocytic characteristics. Moreover, the migration tendency of MCs from fresh donor meniscal tissue into SPX was investigated in an *ex vivo* model.

## 2 Materials and methods

### 2.1 Scaffolds

The commercially available human bone allograft tissues used in this study were provided by the German Institute for Cell and Tissue Replacement (DIZG, gemeinnützige GmbH, Berlin, Germany). All human tissues were acquired from nonprofit tissue recovery partners after informed consent. Grafts were sterilized using a validated, GMP-compliant process and were approved as medicinal products under §21 and §21a of the German Medicinal Products Act. For sterilization, tissues were fully submerged in a validated tissue-preserving sterilization solution (2% peracetic acid, 96% ethanol, water for injection; ratio v/v/v 2/1/1) and incubated with constant agitation at low pressure and room temperature for 4 h (Pruss et al., 2001). Subsequently, tissues were rinsed in a washing process using water for injection. Prior to sterilization, SPX was cut from cancellous bone and freed from bone marrow and lipids. The density was then measured in the mineralized state. A hydrochloric acid-based demineralization step renders SPX suitable for sterilization. Demineralization of cancellous bone leads to elastic and flexible tissue properties. These properties give SPX a sponge like behavior which allows it to act as a shock absorber as well as a scaffold. Meniscal allograft tissue was prepared from human donor knees (Donors: 7 males, aged between 40 and 73 years). After opening the knee capsule, remnants of blood, fat, muscle and connective tissue were removed. Subsequently, the menisci were visually evaluated, and the intact menisci underwent sterilization. Commercially, SPX is available in a freeze-dried state and meniscal allografts are in a deep-frozen state. In this study, the meniscal allografts were thawed and SPX was rehydrated prior to use.

The bovine Collagen Meniscus Implant, CMI (Stryker, MI, United States), served as control scaffold.

The following scaffolds were used:

• SPX with macro pores: SPX (<0.5 g/cm^3^)
• SPX with micro pores: SPX (>0.5 g/cm^3^)
• Meniscus allograft: M-Allo
• Collagen Mencius Implant: CMI

### 2.2 Tissue harvest, cell isolation, and cell culture

Menisci (complete or parts) from 25 donors were harvested: 15 male and 10 female donors (22 patients with gonarthrosis and three patients with post-traumatic gonarthrosis) aged between 56 and 84 years. According to the Pauli classification (Pauli et al., 2011) the menisci used were grade 2–4, without calcium deposits. Approval was obtained from the ethics committee of Jena University Hospital (Umbrella Application: 2018_1158_1-Material, Addendum: 21.08.2020) and written informed consent was obtained from all patients. To isolate meniscal cells (MC), tissues were washed with phosphate-buffered Dulbecco’s saline (DPBS, Sigma-Aldrich, Darmstadt, Germany) and sliced into tiny fragments measuring 1–2 mm³ using a surgical scalpel. They were then treated with Pronase E (Merck, Darmstadt, Germany) for 30 min followed by Collagenase P (Sigma-Aldrich, Darmstadt, Germany) overnight, filtered through a cell strainer, centrifuged, and washed three times with DPBS. Cells were re-suspended in DMEM (PanBiotech, Aidenbach, Germany) supplemented with 10% v/v FBS (Capricorn Scientific, Ebsdorfergrund, Germany), 100 U/mL penicillin and 100 μg/mL streptomycin (Pen/Strep, Gibco, Thermo Fisher Scientific GmbH, Karlsruhe, Germany), counted, and cultured at 37°C in an incubator with 5% CO_2_ for 1–2 weeks until the confluency reached more than 80%. Cells were then trypsinized, harvested, counted, and re-suspended in cryomedium (consisting of 10% DMSO, 20% FBS, and 1% Pen/Strep) for cryopreservation. Cells in passage three to four were used for the following experiments.

### 2.3 Cell differentiation

MCs (4 × 10^4^ per well) were cultured in 24-well plates using four different culture media, namely, normal culture medium (DMEM supplemented with 10% FBS and 1% Pen/Strep), fibroblast medium/DMEM-AS (DMEM supplemented with 10% FBS, 1% Pen/Strep, and 0.17 mM L-Ascorbic acid (Sigma-Aldrich, Darmstadt, Germany), Chondrogenic culture medium (Chondrocyte Basal Medium with 10% FCS supplemented with 10% Chondrocyte growth medium SupplementMix (PromoCell, Heidelberg, Germany, and 1% Pen/Strep), and Osteogenic medium (DMEM supplemented with 0.5 mM L-Ascorbic acid, 10 mM β-Glycerophosphate disodium salt hydrate from Sigma-Aldrich, Darmstadt, Germany, 10 µM Calciumchlorid from Roth, Karlsruhe, Germany, 100 nM Dexamethasone from Sigma-Aldrich, Darmstadt, Germany). Media were changed at days 1, 4, 7, and 10. The viability of cells was measured using PrestoBlue^®^ Cell Viability Reagent (Invitrogen, Life Technologies Corporation, CA, United States) following the manufacturer protocol at days 1, 7, and 14. The old medium was removed from the wells, and 500 µL per well of PrestoBlue^®^ Cell Viability Reagent, diluted 1:10 with the same medium, was added. MCs were incubated for 2 h at 37°C. Measurements were performed in triplicates in a 96-well plate at 570 nm using a plate reader (Epoch, BioTek, CA, United States). The Alkaline Phosphatase (AP) activity of MCs at day 14 was assessed by measuring the absorbance of 4-Nitrophenol at a wavelength of 405 nm, which was released from MCs into the culture supernatants following the incubation with p-nitrophenylphosphate substrate for 30 min at 37°C. Calcium phosphate deposits were assessed by staining the fixed MCs with Alizarin Red S 0.5% (v/v diluted in distilled water) for 10 min at room temperature. Subsequently a solution of 1 g sodium dodecyl sulfate dissolved in 20 mL of 5 mM hydrochloric acid was added, and incubated for 10–20 min to solubilize the stain for quantitation. The absorbance of the solution was measured at 405 nm wavelength using a plate reader (Epoch, BioTek, CA, United States). Human osteosarcoma SAOS-2 cell line served as positive control for the AP-activity measurement and Alizarin Red staining. Cell viability and AP activity experiments were performed in triplicate and repeated with cells from 4 donors. The Alizarin Red staining was performed in duplicate and repeated with cells from 2 donors.

### 2.4 Flow cytometry

Fresh isolated MCs cultured at 37°C in a humidified 5% CO_2_ environment were trypsinised when they reached 80% confluency. MCs (5 × 10^5^) were incubated (30–45 min at room temperature in the dark) with mouse anti-human fluorochrome-conjugated antibodies (from Human Mesenchymal Stem Cell Multi-Color Flow Kit, R&D SYSTEMS, MN, United States) targeting surface markers, namely, CD105/Endoglin-PerCP, CD146/MCAM-CFS, and CD90/Thy1-APC. As a negative marker, a mouse anti-human antibody against CD45 was used. Mouse IgG1 and IgG2A served as isotype controls. Following the incubation, excess antibody was removed by washing the cells with 2 mL of Staining Buffer from the same kit mentioned above. The final cell pellet was resuspended in 200–400 μL of the staining buffer from the kit for flow cytometric analysis using the BD FACSAria™ Fusion (BD Bioscience, Belgium) with FSC/SSC gating and FlowJo v10.8.1 software.

### 2.5 MC on scaffold culture

For each replication, MCs from three different donors in their second to third passages were pooled in equal numbers. SPX (<0.5 g/cm^3^ or >0.5 g/cm^3^), Meniscus Allograft (M-Allo) and CMI underwent shaping into a cylindrical form by employing a surgical biopsy punch with a 5 mm diameter. Samples were placed in a 48-well plate and pretreated overnight in DMEM culture medium supplemented with 10% FBS, 1% Pen/Strep. MCs (4 × 10^4^ per well) suspended in 10 µL culture medium were gently added on top of the scaffolds and the wells were filled with DMEM culture medium supplemented with 10% FBS, 1% Pen/Strep up to 100 µL. The 48-well plate was incubated at 37°C for 1 h, after which 400 µL culture medium was added to each well. After 24 h, the scaffolds were carefully removed from the wells and transferred into a new one. This step was performed to exclude the cells that were not attached to the scaffolds. Cells with grafts were cultured for 7 days with a medium change every 2–3 days. Cells in 2D culture served as a control. Cell viability experiments were performed in triplicate and repeated with 3 pools of cells from 9 donors.

### 2.6 Scaffolds tight-fit in meniscus blocks, *ex vivo* model for partial meniscus repair

Fresh meniscal tissues (4 donors) were cut into wedge-shaped pieces of almost equal size and punched using a 5 mm diameter surgical biopsy punch. Four different scaffolds (SPX <0.05 g/cm^3^, >0.05 g/cm^3^, SPX, M-Allo, and CMI) were tight-fit added into the punched meniscus blocks and placed in 24-well plates filled with culture medium (DMEM supplemented with 10% FBS, 1% Pen/Strep). Meniscus cylinders taken from the biopsy punches of the fresh menisci were also cultured in the same medium as a control. The cultivation lasted for 7 or 28 days, with the medium being changed every 2–3 days. Cell viability experiments were performed in triplicate.

### 2.7 Enzyme-linked immunosorbent assay (ELISA)

The concentrations of human Pro-Collagen I α 2 (Pro-Col1) and human Pro-Collagen II α 1 (Pro-Col2) in supernatants from MCs cultured in different differentiation media (from day 14 post cultivation) and MCs cultured with scaffolds (from days 1 and 7 post cultivation) were measured using the Human Pro-Collagen I alpha 1 DuoSet ELISA kit, Human Pro-Collagen II DuoSet ELISA kit and DuoSet ELISA Ancillary Reagent Kit 2 (R&D SYSTEMS, MN, United States). The procedure of the assay was conducted according to the manufacturer’s instructions.

### 2.8 RNA extraction, cDNA synthesis, and quantitative real-time polymerase chain reaction (qPCR)

RNA from monolayer-cultured MCs was extracted using the RNeasy Plus Mini Kit (Qiagen, Hilden, Germany). MCs cells cultured on scaffolds were extracted with a combination of the Trizol method (Invitrogen, Life Technologies Corporation, CA, United States) and chloroform extraction, followed by using RNeasy Plus Mini Kit. The concentration of the RNA was determined by measuring the absorbance of a diluted sample of the RNA at 260 and 280 nm using a NanoDrop™ 2000c (Thermo Fisher Scientific GmbH, Karlsruhe, Germany) after re-suspending it in RNase-free water. The RNA was placed in liquid nitrogen and stored at −80°C. Reverse transcription of 100 ng of RNA from each sample into cDNA was conducted with the qScript^®^ cDNA SuperMix reverse transcription kit (from Quantabio, Beverly, MA, United States) using a polymerase chain reaction (PCR) method, as per the manufacturer’s protocol.

The gene expression levels of Beta actin (*ACTB*), Glyceraldehyde 3-phosphate dehydrogenase (*GAPDH*), Aggrecan (*ACAN*), Runt-related transcription factor 2 (*RUNX2*), Alkaline phosphatase (*ALP*), Collagen Type I Alpha 2 (*COL1*), Collagen Type II Alpha 1 (*COL2*), SRY-Box Transcription Factor 9 (*SOX9*) were determined using the PerfeCTa SYBR Green SuperMix kit (Quantabio, Beverly, MA, United States) and human-specific primers from TIB MOLBIOL Syntheselabor GmbH, Berlin, Germany (Table 1). The qRT-PCR was performed on Rotor-Gene Q (Qiagen, Hilden, Germany) according to the manufacturer’s instructions. Relative expression was calculated according to the 2^−ΔΔCT^ method, and results were normalized to the arithmetic average of *ACTB* and *GAPDH* expression.

**TABLE 1:** List of primer sequences for the target genes used for qRT-PCR.

Target genes forward and reverse primer sequences reported by TIB MOLBIOL
*ACTB* _ Forward 5‘-AAG​CCA​CCC​CAC​TTC​TCT​C-3‘ Reverse 5‘-GCT​ATC​ACC​TCC​CCT​GTG-3‘
*GAPDH* _ Forward 5‘-GAA​GGT​GAA​GGT​CGG​AGT​C-3‘ Reverse 5‘-TCG​CTC​CTG​GAA​GAT​GGT​G-3‘
*ACAN* _ Forward 5‘-ACT​CTG​GGT​TTT​CGT​GAC​TCT-3‘ Reverse 5‘-ACA​CTC​AGC​GAG​TTG​TCA​TG-3‘
*RUNX2* _ Forward 5‘-TGG​TTA​CTG​TCA​TGG​CGG​G-3‘ Reverse 5‘-TCT​CAG​ATC​GTT​GAA​CCT​TGC-3‘
*ALP* _ Forward 5‘-ACC​ACC​ACG​AGA​GTG​AAC​C-3‘ Reverse 5‘-CGT​TGT​CTG​AGT​ACC​AGT​CC-3‘
*COL1* _ Forward 5‘-GGG​CCA​AGA​CGA​AGA​CAT​C-3‘ Reverse 5‘-CAG​ATC​ACG​TCA​TCG​CAC​AAC-3‘
*COL2* _ Forward 5‘-TGG​ACG​CCA​TGA​AGG​TTT​TCT-3‘ Reverse 5‘-TGG​GAG​CCA​GAT​TGT​CAT​C-3‘
*SOX9* _ Forward 5‘-AGC​GAA​CGC​ACA​TCA​AGA​C-3‘ Reverse 5‘-CTG​TAG​GCG​ATC​TGT​TGG-3‘

### 2.9 Histology

After 7 or 28 days of MC culture on scaffolds or within the meniscus, samples were fixed in 4% Paraformaldehyde for 24–48 h and were paraffin embedded. Samples were cut into 5 μm sections. The samples were then stained with DAPI (Thermo Fisher Scientific GmbH, Karlsruhe, Germany) and assessed under the microscope (Keyence BZ-810 Fluorescence Microscope). Experiments were replicated four times. For quantitative analysis, two slides per sample were used and all stained cells within the scaffolds were counted.

### 2.10 Statistical analyses

All data were presented as median with interquartile range (IQR) or mean ± standard error of the mean (SEM). Due to the small sample size and the non-parametric distribution of the data, statistical differences between samples were analyzed using nonparametric One-Way ANOVA test with GraphPad Prism software version 9.3.1 (San Diego, CA, United States). The effect size was calculated using Cohn´s d (Cohen, 1988) and for all statistically significant differences this was d > 0.8, meaning a large effect.

## 3 Results

### 3.1 Viability of MCs cultured in different culture media (differentiation media)

**FIGURE 1:**
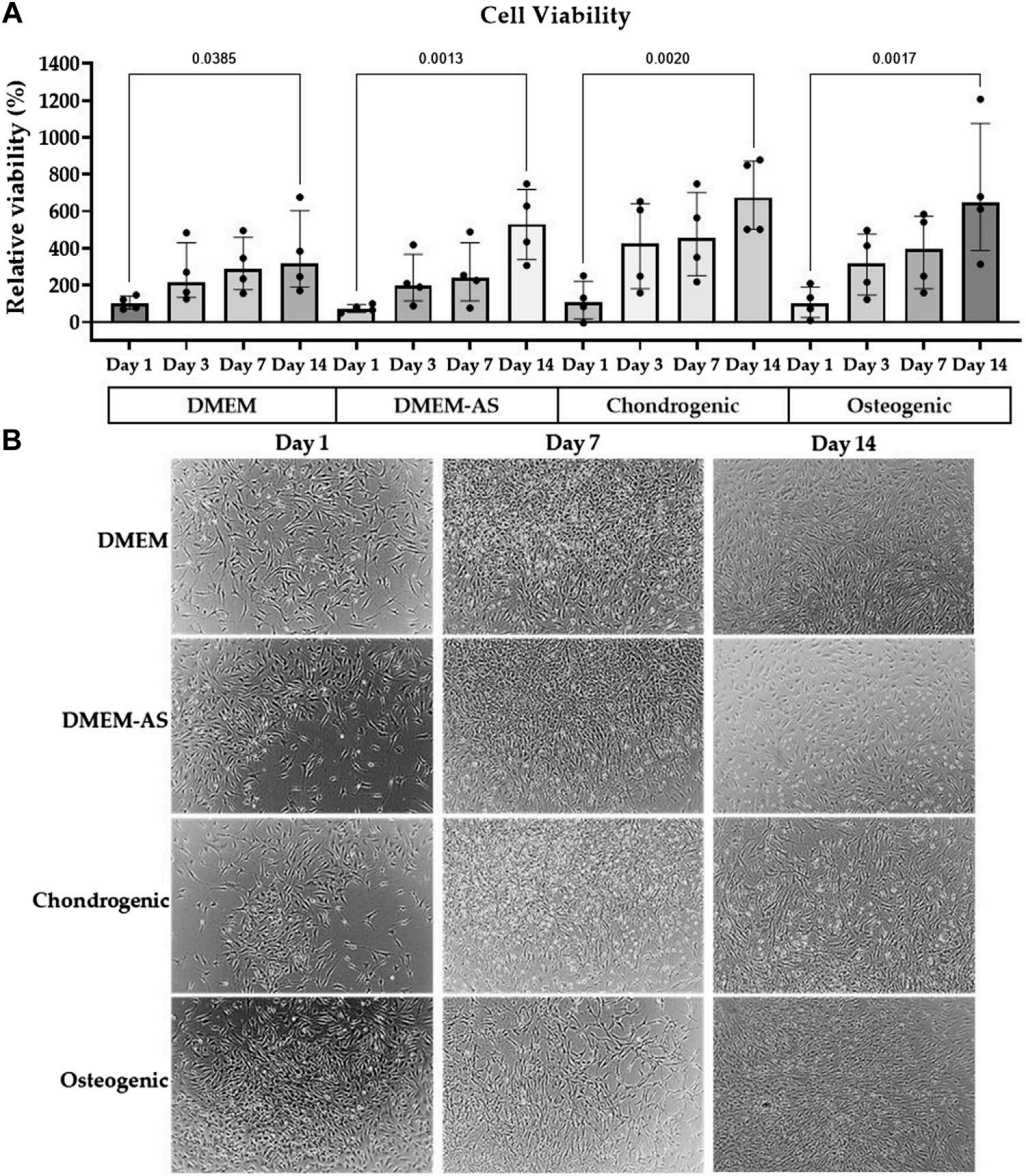
MCs viability and morphology in differentiation media. The viability of MCs increased over time when cultured in various media **(A)**, and their morphology stayed consistent **(B)**. The bar graph is based on the results of four experiments (n = 4) with the median (IQR) displayed in the bars.

The viability assay, which was also used as an indirect measure of cell number, showed the viability/cell number of MCs, cultured in four different media (DMEM, DMEM-AS, chondrogenic and osteogenic) at four time points (day 1, 3, 7 and 14, Figure 1A). Viability values were normalized to the viability of MCs cultured in DMEM at day 1. The results showed that MCs grew at a similar rate. A significant increase in MCs viability was observed in all four media at day 14 compared to day 1 (*p* ≤ 0.0385, see figure for exact *p*-values). Moreover, the morphology of MCs in differentiation media was visualized by light microscopy at different time intervals (Figure 1B). No morphological changes were observed over time.

### 3.2 Osteogenic differentiation

**FIGURE 2:**
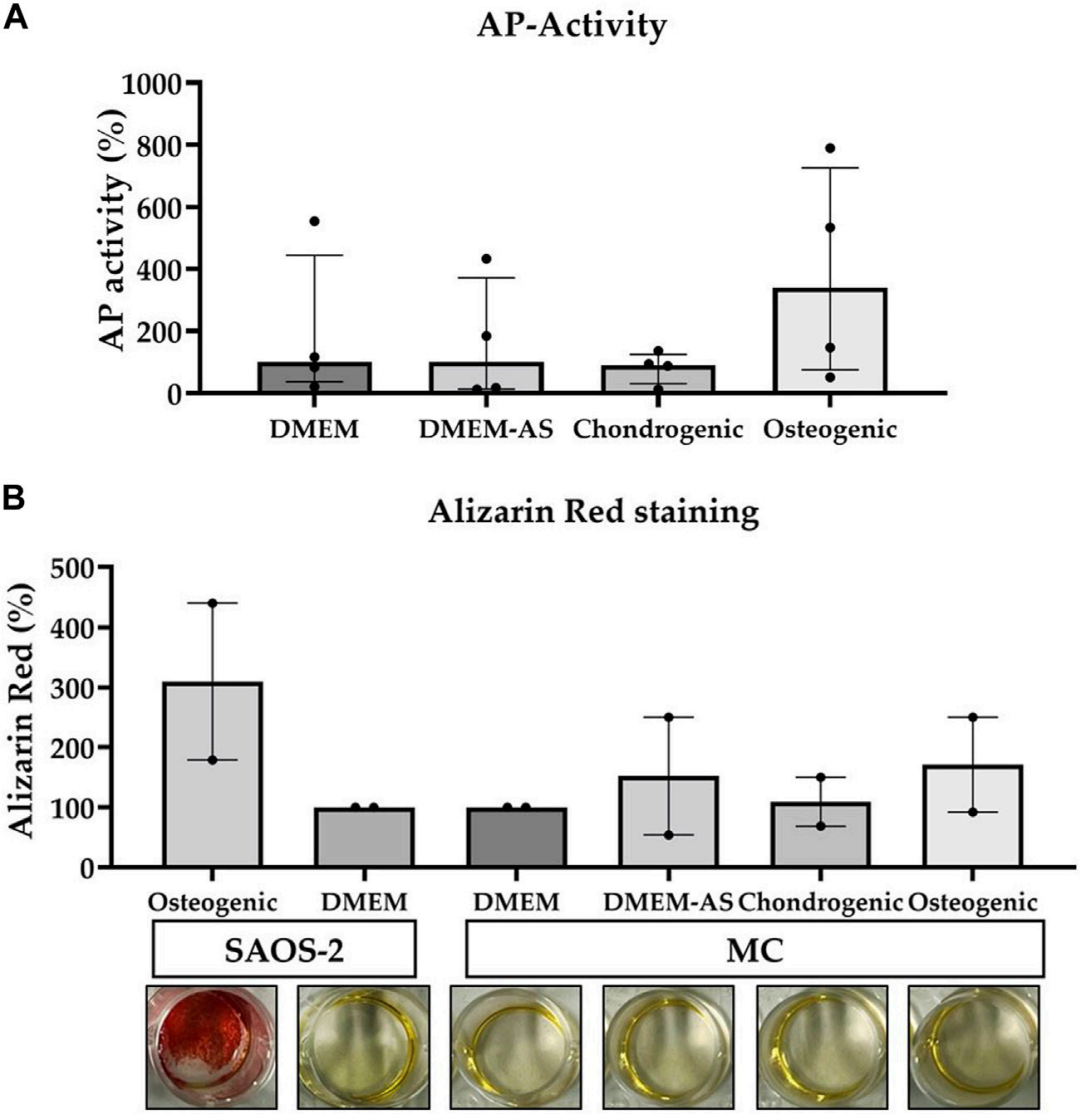
The AP-activity of MCs and their production of calcium phosphate deposits measured when cultured in various media. Osteogenic medium resulted in a relatively higher AP-activity of MCs compared to other media **(A)**. The calcium phosphate deposits produced by MCs cultured in the different media were less than those produced by SAOS-2 cells in osteogenic medium **(B)**. The data is based on four (n = 4) and two (n = 2) experiments respectively. The bars represent median (IQR). AP: alkaline phosphatase, MC: meniscal cell.

MCs cultured in osteogenic medium had a relatively higher alkaline phosphatase (AP) activity than the MCs cultured in other media at day 14 (Figure 2A). Alizarin Red staining showed that calcium phosphate deposition (evidence of osteogenic differentiation) of SAOS-2 cells cultured in osteogenic medium was higher than that of MCs cultured in DMEM, DMEM-AS, chondrogenic, and osteogenic media at day 14 (Figure 2B). In contrast, MCs exhibited no major difference in the amount of calcium phosphate deposits when cultured in different media.

### 3.3 Pro-Col1 and 2 production by MCs cultured in differentiation media and MCs surface markers

**FIGURE 3:**
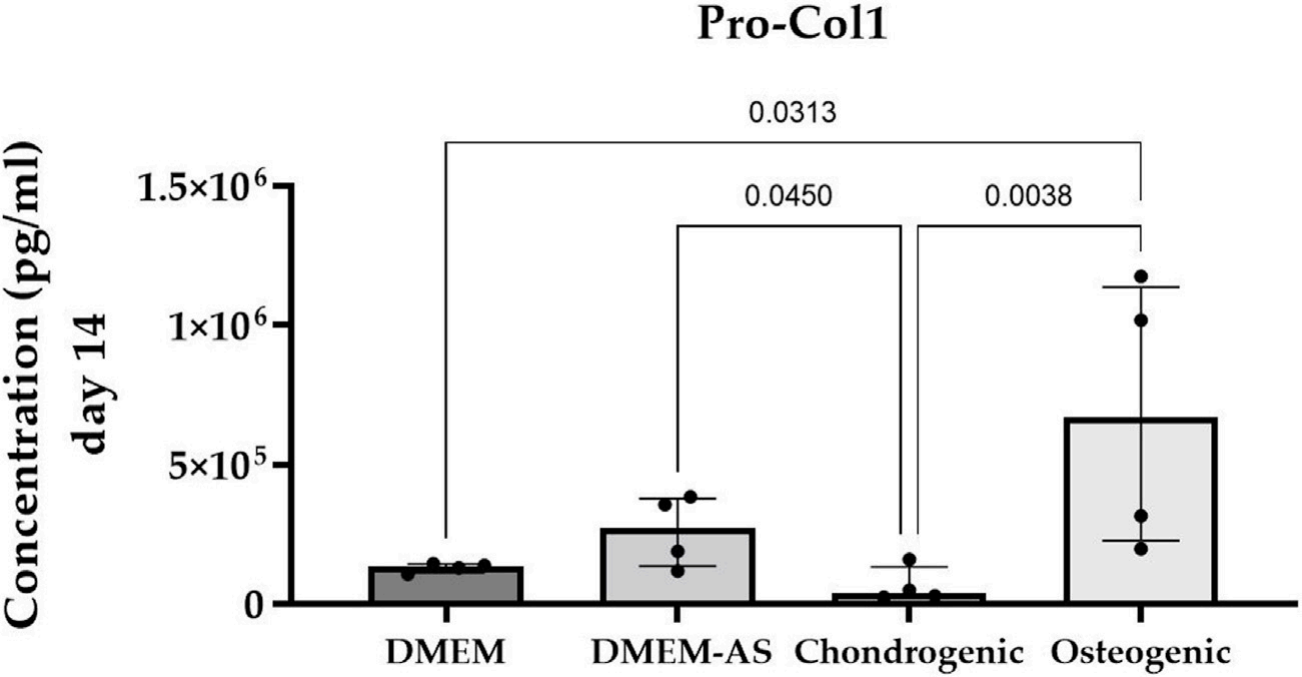
Cultivation of MCs in osteogenic medium resulted in higher production of Pro-Col1 compared with MCs cultured in other media. Bar graph showing the median (IQR) of results from four experiments (n = 4).

MCs cultured in osteogenic medium for 14 days produced significantly more Pro-Col1 than MCs cultured in DMEM and chondrogenic medium (*p* ≤ 0.0313, exact *p*-values see Figure 3). Culturing MCs in DMEM-AS resulted in greater amounts of Pro-Col1 than MCs cultured in chondrogenic medium (*p* = 0.045). The levels of Pro-Col2 were below the detection limit in all samples as determined by ELISA. Flow cytometric analysis revealed that CD90 was expressed on the majority of cells (88.1%), whereas CD105 and CD146 were expressed on only 7.5% and 0.7% of cells, respectively (surface markers for mesenchymal stromal cells). CD 45 (marker for nucleated hematopoietic cells) was expressed on 0.4% of cells (n = 4).

### 3.4 Expression of osteogenic and chondrogenic genes in MCs cultured in differentiation media

**FIGURE 4:**
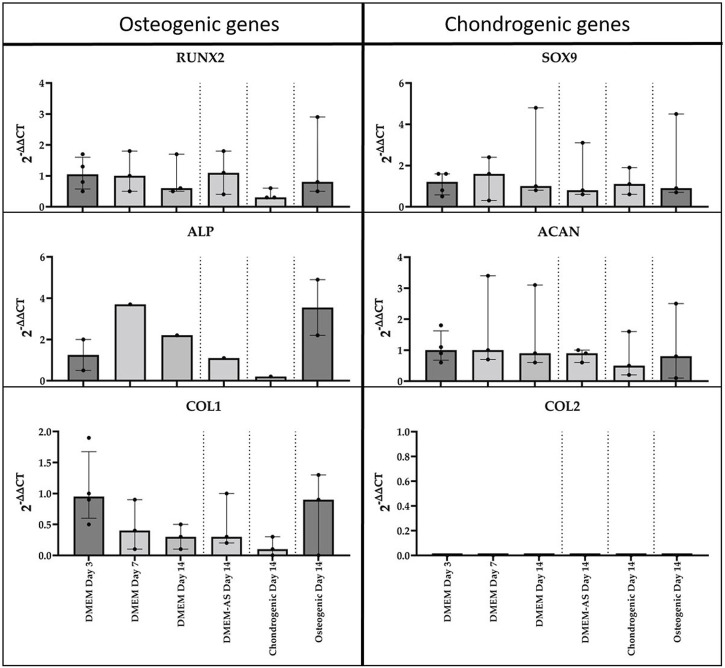
qPCR analysis of cDNA of RUNX2, ALP, COL1, SOX9, ACAN, and COL2 genes in monolayer MCs culture in various media (n = 3). Data all presented as median (IQR).

The qPCR analysis of MCs cultured in differentiation media revealed no notable alterations in the expression of osteogenic and chondrogenic genes after 14 days of culture (Figure 4). It should be mentioned that not all genes were expressed in all samples and that the results showed a high variation. *COL2* expression was not detected.

### 3.5 Viability of MCs cultured on scaffolds and their Pro-Col1 and 2 production levels

**FIGURE 5:**
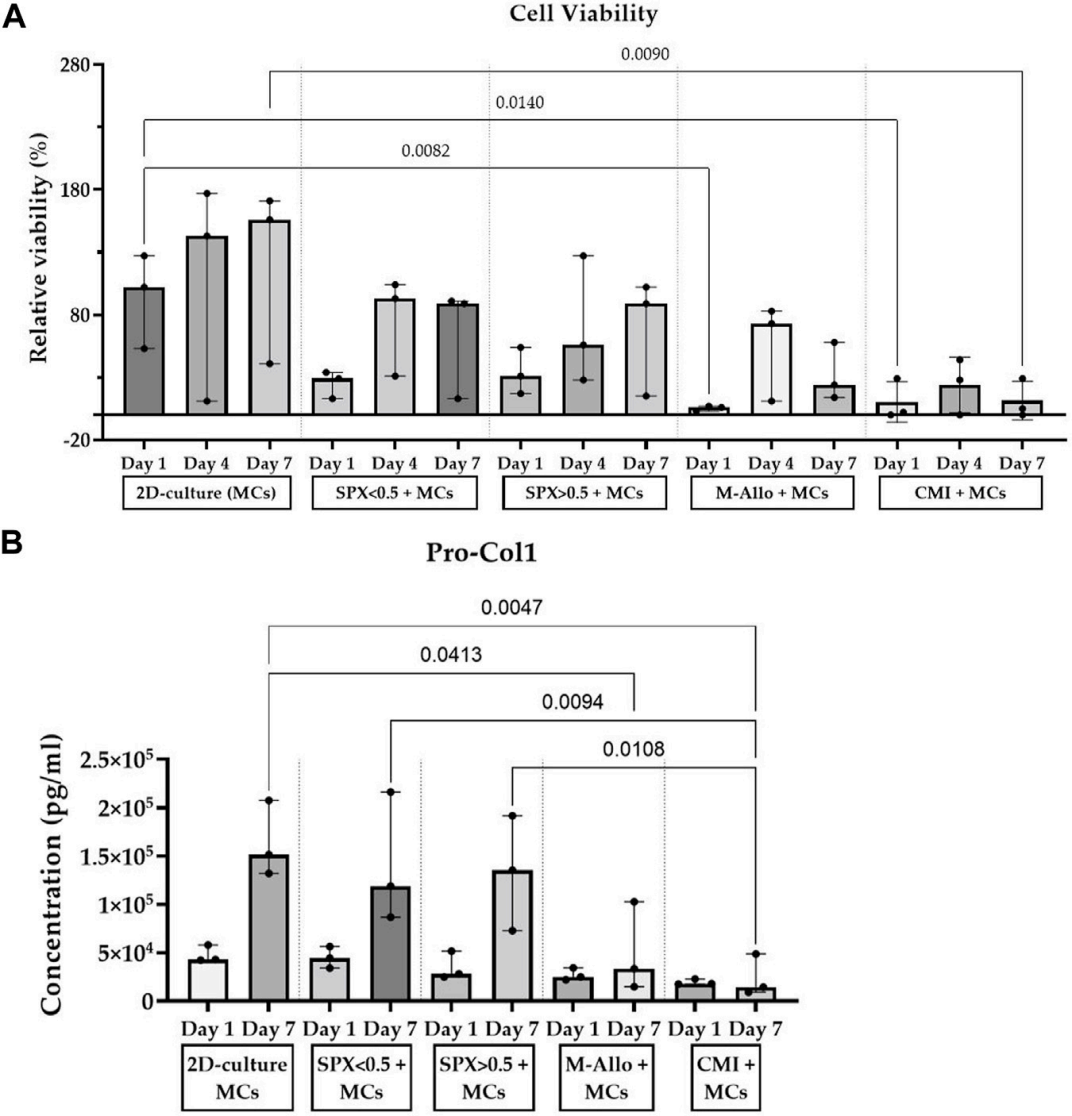
**(A)** Viability results show an increase in MCs cultured on SPX and in 2D culture for 7 days. **(B)** MCs cultured on SPX produced higher amounts of Pro-Col1 than MCs cultured on CMI. Viability values are normalized to the viability of 2D-culture MCs at day 1, n = 3, bars represent median (IQR). SPX: spongioflex^®^, M-Allo: meniscus allograft, CMI: collagen meniscus implant, Col1: collagen 1, MCs: meniscal cells.

The viability/cell number of MCs cultured in 2D, on SPX with micropores, SPX with macropores, M-Allo, and CMI at different time intervals (days 1, 4, and 7) indicated a time-dependent increase in the viability of MCs cultured in 2D and on both SPX porosities (Figure 5A). In contrast, cells cultured on M-Allo and CMI displayed reduced viability by day 7. On day 1, the viability/cell number of 2D cultured MCs was significantly higher than that of MCs cultured on M-Allo, and CMI scaffolds (*p* = 0.0082 and *p* = 0.009, respectively). Only 16% ± 2% (mean ± SEM) viability was detected when measuring the viability of the cells in the “transferred” wells (the wells in which the scaffolds had been inserted into on day 0) of the scaffolds on day 1 (n = 3). Meaning, approximately 16% of cells were lost during the transfer of the scaffold between day 0 and day 1. Furthermore, the viability of 2D-cultured MCs was significantly higher than the viability of MCs cultured on CMI (day 7; *p* = 0.014). The Pro-Col1 concentration in the supernatants of MCs cultured in 2D conditions and on SPX (both porosities) was higher at day 7 compared with day 1, although the differences were not statistically significant (Figure 5B). Moreover, Pro-Col1 levels in 2D cultured MCs were significantly higher than those of MCs cultured on M-Allo and CMI at day 7 (exact *p*-values see figure). In addition, the levels of Pro-Col1 in the supernatant of micro- and macro-pores SPX were also significantly higher than in the supernatant of MCs cultured on CMI. In all samples, the Pro-Col2 levels were below the detection limit.

### 3.6 Expression of osteogenic and chondrogenic genes in MCs cultured on scaffolds

**FIGURE 6:**
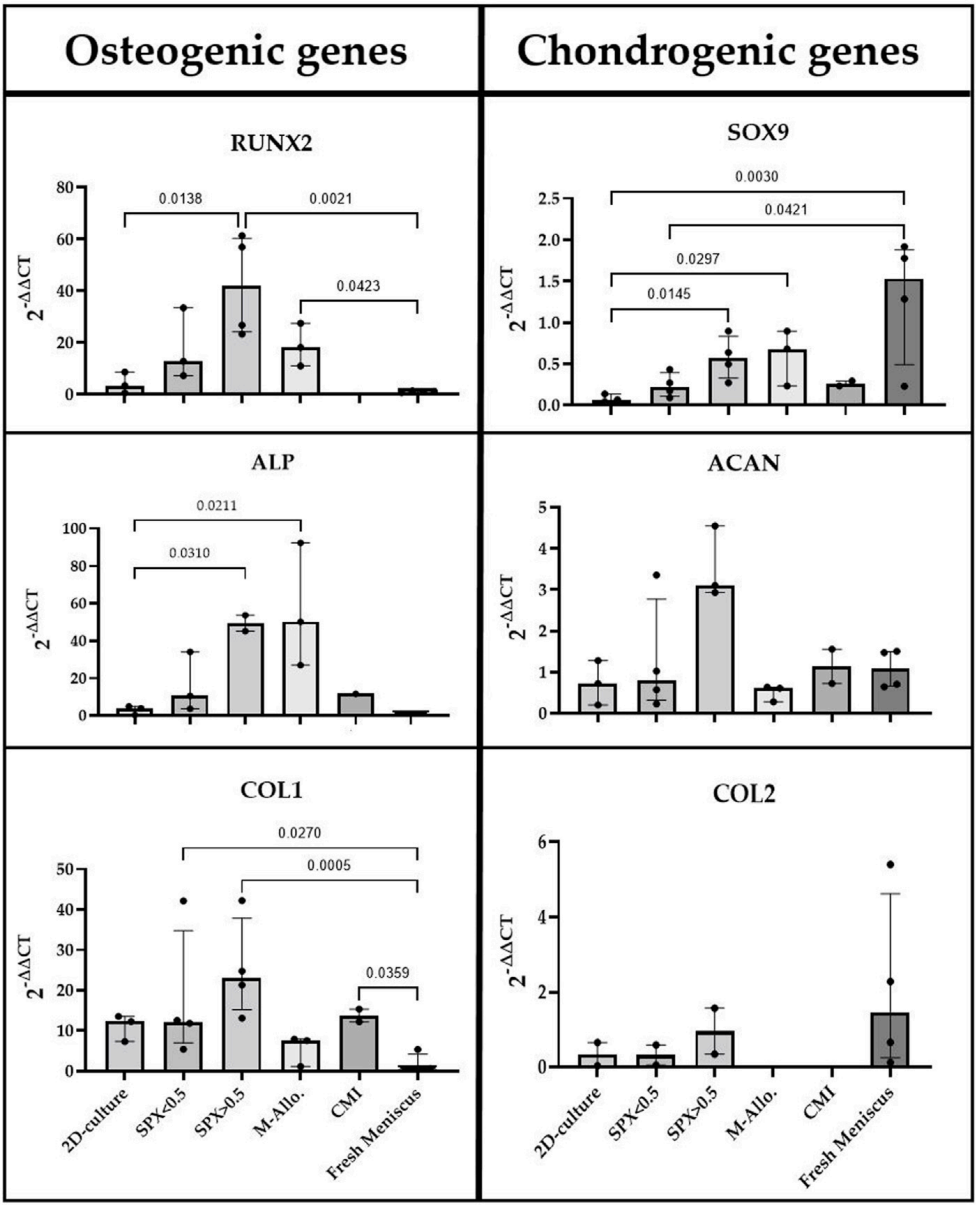
qPCR analysis of cDNA of RUNX2, ALP, COL1, SOX9, ACAN, and COL2 genes in monolayer MCs culture, MCs in fresh meniscus, and MCs cultured on SPX, M-Allo, and CMI (n = 3–5). Data all presented as median (IQR). SPX: spongioflex^®^, M-Allo: meniscus allograft, CMI: collagen meniscus implant.

The expression of osteogenic and chondrogenic related genes showed that MCs cultured on micro-pores SPX have the highest expression of analyzed genes (e.g., *RUNX2, ALP* and *SOX9* significantly higher compared to 2D culture, see Figure 6 for exact *p*-values). The expression level of *Col1* in MCs on SPX (both porosities) was significantly higher than in fresh meniscus. *SOX9* expression level was highest in fresh meniscus. Low expression values or no gene expression was observed when MCs were cultured on CMI. This is due to the low cell number and RNA values in these cultures. Further details on the gene expression and significant differences are shown in Figure 6.

### 3.7 Histological DAPI staining of MCs cultured on scaffolds

**FIGURE 7:**
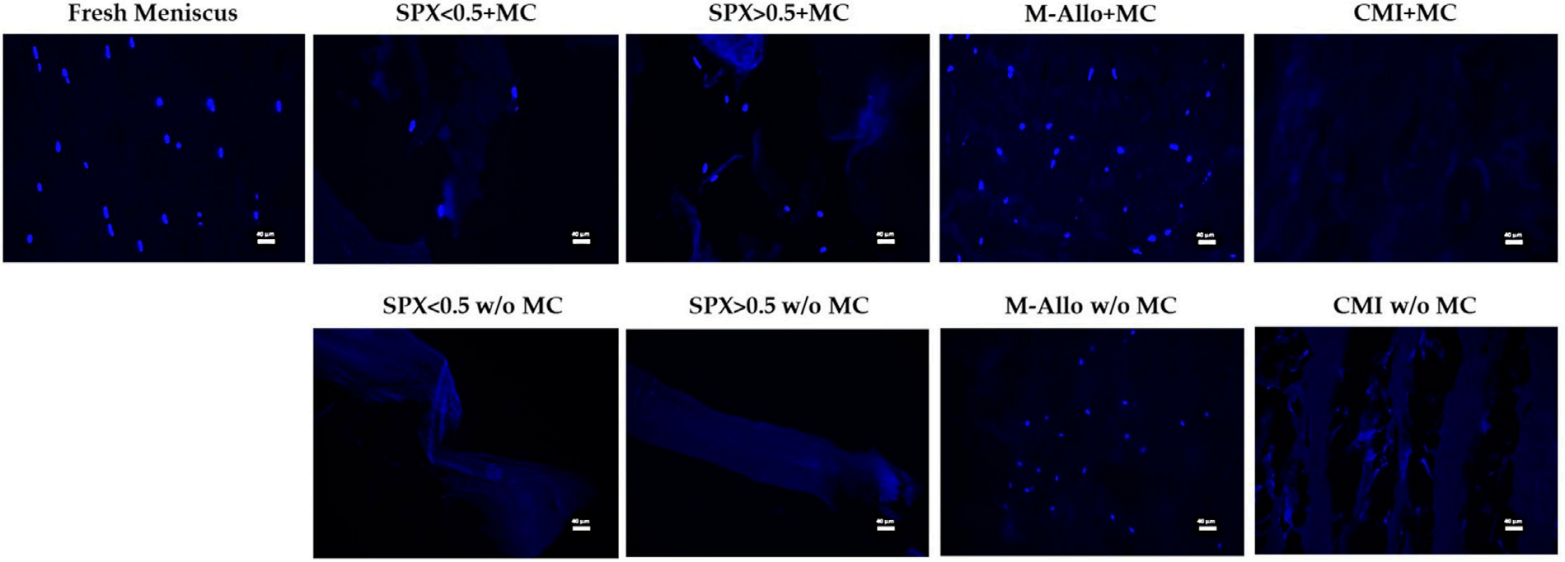
Fluorescent images of MCs in fresh meniscus and cultured on different scaffolds stained with DAPI. Scale bars in the images equal 40 µm. SPX: spongioflex^®^, M-Allo: meniscus allograft, CMI: collagen meniscus implant, MC: meniscal cell, w/o: without.

DAPI staining of paraffin-embedded sections of various scaffolds cultured with MCs showed the presence of MCs in SPX (both porosities) (Figure 7). No cells were seen in CMI with MCs after 7 days of cultivation. It was not possible to distinguish whether MCs grew on M-Allo because the DAPI staining was seen in M-Allo without culture with MCs.

### 3.8 Viability of meniscal tissues cultured with scaffolds (*ex vivo* meniscus model)

**FIGURE 8:**
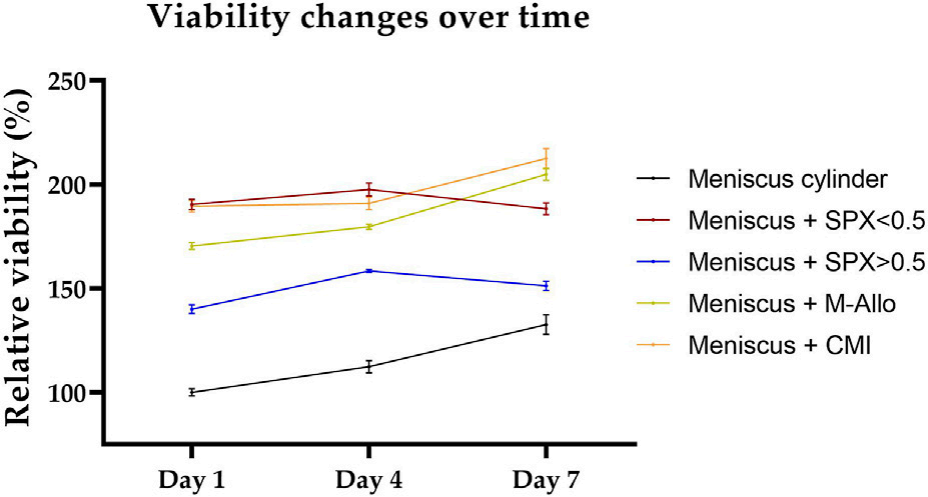
Viability of meniscus blocks cultured with different scaffolds over a period of 7 days in DMEM culture medium. Viability values are normalized to the viability of the meniscal cylinder at day 1. n = 4, bars represent mean ± SEM. SPX: spongioflex^®^, M-Allo: meniscus allograft, CMI: collagen meniscus implant.

The viability of fresh human meniscal tissue blocks (cultured with micro-pores SPX, macro-pores SPX, M-Allo, and CMI) was measured using the PrestoBlue^®^ viability assay. Although the viability values were not directly comparable due to the differences in size and cell numbers of the fresh meniscus blocks, the findings suggest that, at least within a 7-day timeframe, the scaffolds do not exhibit cytotoxic effects on the meniscal cells (Figure 8). This was determined by observing that the viability of the tissue blocks was not altered over the 7 days in culture.

### 3.9 Expression of osteogenic and chondrogenic genes in *ex vivo* meniscus with scaffolds

**FIGURE 9:**
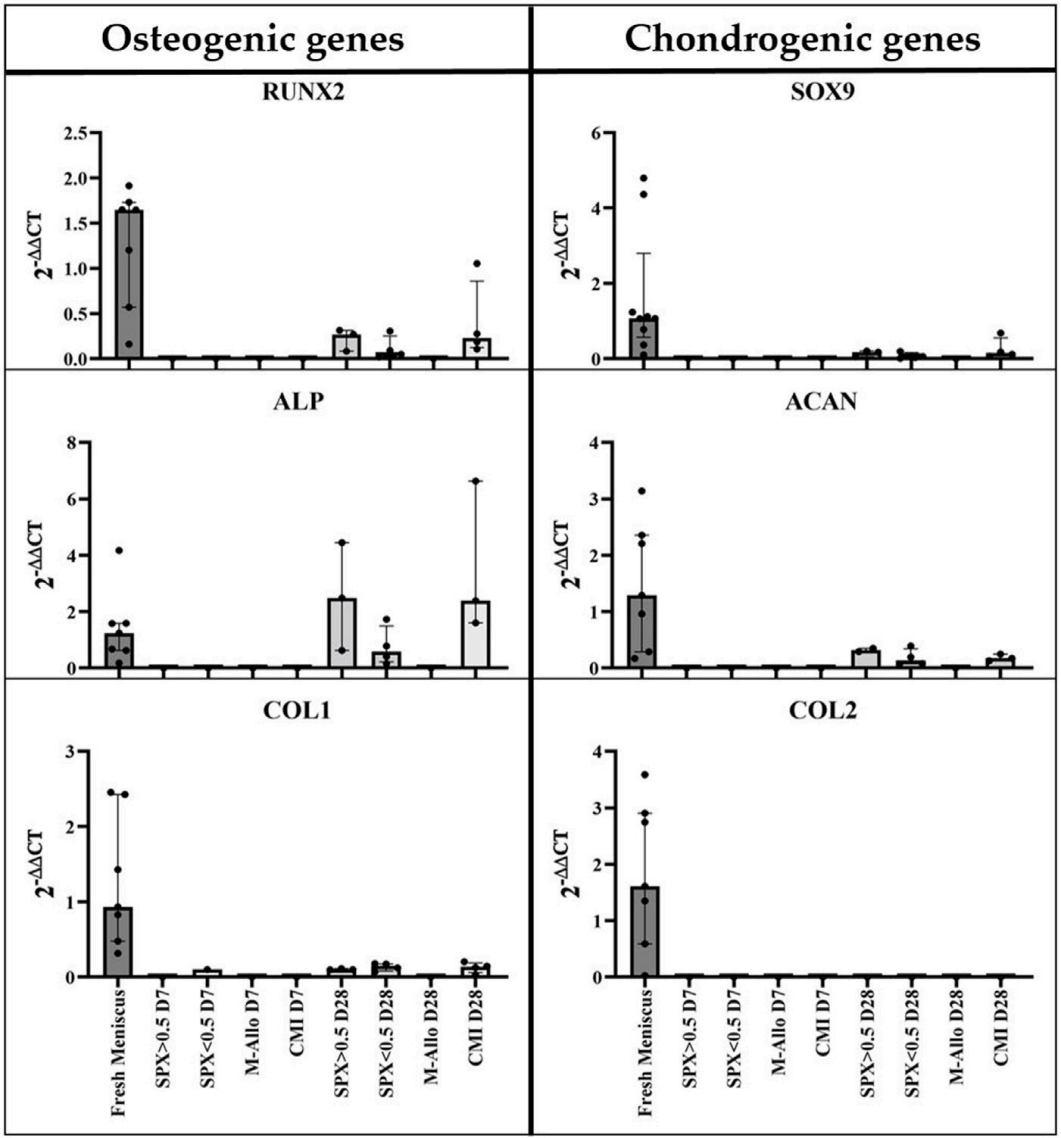
qPCR analysis of cDNA of RUNX2, ALP, COL1, SOX9, ACAN, and COL2 genes in MCs in fresh meniscus and in migrated MCs into SPX, M-Allo, and CMI (n = 7 for fresh meniscus and n = two to five for scaffold). Data all presented as median (IQR). SPX: spongioflex^®^, M-Allo: meniscus allograft, CMI: collagen meniscus implant.

To analyze the gene expression, the scaffolds were removed from the menisci and the RNA was separately isolated. Osteogenic and chondrogenic genes were expressed in the cultured fresh meniscal tissue (Figure 9). The expression of these genes was found to be low or not detectable in the separated scaffold samples after 7 days of culture, partially due to the low amount of isolated RNA. On the other hand, the expression of *RUNX2*, *ALP*, *SOX9*, and *ACAN* in migrated cells in SPX (both porosities) and CMI after 28 days of co-cultivation was higher than that after 7 days of cultivation.

### 3.10 Histological DAPI staining of *ex vivo* meniscus with scaffolds

**FIGURE 10:**
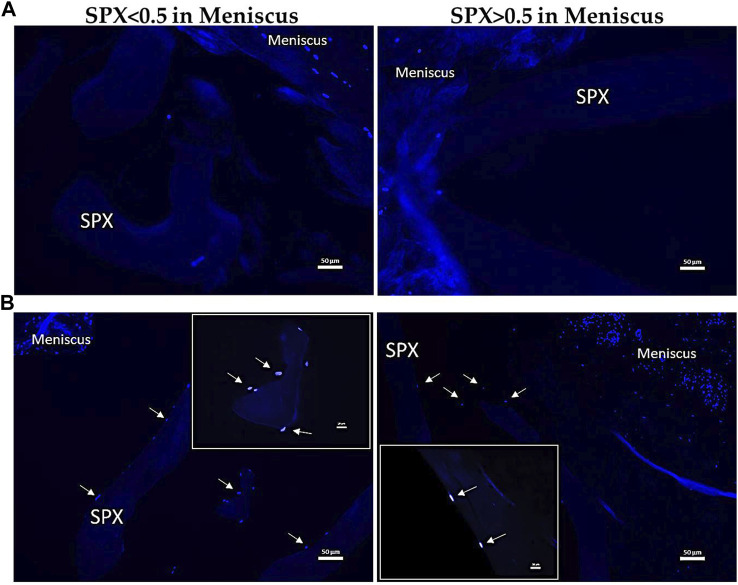
Fluorescent images of MCs in meniscus blocks containing SPX for 7 **(A)** and 28 **(B)** days stained with DAPI. The arrows indicate the migration of MCs from the surrounding meniscal tissue. Scale bars in the images equal 50 µm in A and B respectively. SPX: spongioflex^®^.

DAPI staining demonstrated that not until 28 days after culturing the scaffolds in the meniscus, MCs migrate from the meniscus to the SPX. (Figure 10). Quantification of the number of cells that migrated into the scaffolds showed ingrowth of cells in SPX (both micro- and macro-pores) and in CMI (60 ± 46 cells for macro-pores SPX, 57 ± 53 cells for micro-pores SPX, and 53 ± 19 for CMI, n = 4).

## 4 Discussion

The meniscus has a low regeneration potential and the current regenerative therapies are limited, often resulting in an unsatisfactory treatment or, as a last resort, to the resection of the meniscus. This *in vitro* and *ex vivo* study aimed to investigate a possible grafting material for meniscus regeneration. Human meniscal cells or meniscal tissue was cultured with different grafting materials to invesitgate cell ingrowth and differentiation. Two types of human allografts were used: SPX (demineralized cancellous bone) with different porosities (porosity approximated by the weight, macro-pores: <0.5 g/cm³ and micro-pores: >0.5 g/cm³) and M-Allo. CMI served as the control material, as it is also made of collagen. Cells cultured on SPX showed an increase in viability over 7 days, whereas those on M-Allo or CMI were less viable. The synthesis of the extracellular matrix protein collagen I was comparable between cells in 2D and cells on SPX. Cell ingrowth was only detectable for cells cultured on SPX. *Ex vivo* organ culture showed that cells had not grown into the scaffolds after 7 days, resulting in no detectable gene expression. However, MCs were able to migrate from the meniscal tissue to the SPX within 28 days. Gene expression analysis showed that the expression of *RUNX2*, *ALP*, *SOX9*, and *ACAN* increased in cells that migrated from meniscal tissue to SPX (both micro- and macro-pores) and CMI after 28 days of co-cultivation. The porous scaffold provided attractive conditions for cell growth within 7 days, but a longer time (28 days) was required to stimulate cell migration from the surrounding tissue in an *ex vivo* culture.

The cells used in this study were isolated from human donor menisci (complete or parts). The menisci were harvested from patients with gonarthrosis and the menisci were grade 2–4 without calcium deposits based on the Pauli classification (Pauli et al., 2011). This resulted in an inhomogeneous cell population as seen in the different areas of the meniscus. Verdonk et al. published one of the first studies on human meniscal cell characteristics and demonstrated their distinct phenotype and improved fibrochondogenic properties when cultured in 3D (Verdonk et al., 2005). Using flow cytometry, they characterized human meniscal cells and the cell-associated extracellular matrix and distinguished different cell types. Depending on the donor, they found a high variability of collagen I, II and aggrecan. A high variability in gene expression was also seen in other studies (Liang et al., 2017; Crawford et al., 2020) and might further be explained by the inhomogeneous structure of the meniscus. According to Zheng et al., different cell sources such as bone marrow mesenchymal stem cells, adipose-derived stem cells, and articular chondrocytes demonstrate diverse proliferative behaviors, distinct biochemical and biomechanical attributes, and varying gene expression profiles (Zheng et al., 2023). The variations in gene expression seen in these experiments can therefore be explained by the different donors and different regions of the meniscus. In addition, the natural structural differences in the meniscus with varying proportions of different cell types in distinct regions could influence our results. As a limitation of this study, no mechanical stimulation of the constructs was performed. Mechanical loading has a proven role in enhancing fibrochondrocyte differentiation and might affect the cells in the scaffolds as summarized in a review by Ma et al. (Ma et al., 2022a). It impacts the cell behavior and especially the gene expression, particularly of extracellular matrix proteins. For instance, it has been demonstrated to enhance the differentiation of fibrochondrocytes derived from Bone Marrow Mesenchymal Stem cells (BMSCs) in fibrin constructs, leading to an upregulation of COL1 expression (Connelly et al., 2010). Furthermore, studies have indicated that mechanical stimulation can increase the levels of aggrecan, COL1, and COL2 in chondrocytes (Shadi et al., 2022). Sex-specific differences were seen in the reaction of engineered menisci depending in the mechanical stimuli (Ma et al., 2022b).

Liang et al. investigated the expression of osteogenic genes after 21 days of culturing meniscus fibrochondrocytes, discovering no differences in the modified culturing environment. Similarly, their cells were negative for the Alizarin red stain, as was the case in this study (Liang et al., 2017). In contrast, Fu et al. found an osteogenic differentiation of meniscal cells 14 days after culturing under osteogenic conditions (Fu et al., 2016). Both studies used cells at passage 3 but with different time points and Liang et al. also added TGFβ1 and FGF-2 to the medium. Culturing the MCs for 7 days in osteogenic medium resulted in no osteogenic differentiation, although the control cells (SAOS2) showed a strong Alizarin red stain in this study. Osteogenic differentiation is an important aspect, and the reason for the different osteogenic gene expression should be investigated further, as an osteogenic differentiation of the cells within the meniscus graft would be an unwanted effect. Furthermore, the lack of COL2 expression in MCs cultured in the different media might be due to the cultivation period being too short.

Analyzing cell surface markers showed that almost all cells isolated from the meniscus expressed CD90, but only a few cells were positive for CD105 or CD146. Recent studies investigated the phenotype of meniscal cells using flow cytometry and single cell sequencing. They found regional differences with high amounts of CD90-positive cells and up to seven independent cell clusters (Sun et al., 2020; Wang et al., 2022). The low amount of CD146-positive cells in this study might indicate towards a special phenotype found in degenerated menisci: degenerated meniscus progenitor cells (DegP) (Sun et al., 2020). Interestingly, Verdonk et al. found a low amount of CD105 and CD44 positive cells but an increase in this surface marker when cells were cultured in alginate (Verdonk et al., 2005). Cellular properties are different depending on the localization, the degeneration of the meniscus (Fu et al., 2022), and culture conditions (fibronectin coating, physioxia vs. hyperoxia) that can be modified to enhance the progenitor phenotype of isolated meniscal cells (Pattappa et al., 2022). The cells used in this study were isolated from the entire meniscal tissue obtained without separation of different meniscal areas and further isolation of the progenitor cells. Moreover, the meniscal tissue was obtained from surgery and the menisci showed signs of alterations. Attention was paid to using only tissue areas with fewer alterations for cell isolation and the *ex vivo* meniscus model, but it cannot be excluded that the cells were affected and do not fully resemble healthy meniscal cells. The variation in the results can be explained by the variations described for affected meniscal tissue (Bradley et al., 2023). This would also be the case in the clinical situation where the scaffold would be implanted into injured meniscus at different regions. However, this approach might explain the high variation in the data as the cell populations might be very inhomogeneous.

The majority of the extracellular matrix of a native meniscal tissue is composed mainly of collagen I and a smaller amount of collagen II and glycosaminoglycans, which serve as a substrate for cell migration and proliferation (Chen et al., 2015; Lyons et al., 2019; Ruprecht et al., 2019; Zhong et al., 2020; Bansal et al., 2021). Biocompatible materials have been shown to facilitate cell adhesion, promote cell growth, and stimulate collagen deposition (Murakami et al., 2017; Baek et al., 2019). Despite large interconnected pores of the porous SPX, only a few of the suspended cells were actually able to pass through, and the majority of the cells remained stuck in the scaffolds. Nevertheless, the cell viability assay indicated that not all of the trapped cells were able to adhere to the inner surfaces of the scaffolds and were possibly unable to form the intercellular connections and extracellular matrix necessary for survival.

Our results showed that despite many of the cells being able to adhere to the scaffolds on day 0, a proportion died and were consequently removed from the scaffolds. This was likely due to the increased stress caused by the transfer of the scaffolds into a new well and the medium exchange procedure. Additionally, the cells were not able to produce an adequate amount of extracellular matrix within 24 h of seeding. In this study, we demonstrated that as opposed to M-Allo and CMI, SPX also promotes meniscal cell growth and proliferation and stimulates the synthesis of the extracellular matrix protein collagen I. One possible explanation for the lack of cell growth on M-Allo could be the high density of this scaffold, which prevented the cells from initially penetrating inside. Previous studies have also reported high collagen synthesis when different types of stem cells were cultured on biocompatible scaffolds, including bone marrow-derived mesenchymal stem cells, primary MCs, and peripheral blood-derived mesenchymal stem cells (Lyons et al., 2019; Mao et al., 2022; Barceló et al., 2023). The ability of these scaffolds to promote collagen I production is an important finding that could have significant clinical implications. These results suggest that biocompatible materials might be a promising approach for meniscal tissue development and regeneration.

Culturing MCs on SPX with low porosity appears to enhance differentiation of the cells into an osteogenic phenotype, as might be expected from the upregulation of the ALP and RUNX2 genes. However, the expression of the ACAN gene, which is considered a chondrogen-specific gene in these cells, was also relatively upregulated. One possible explanation for the upregulation of osteogenic genes could be the influence of scaffold architecture and pore size on the cells. Studies have reported the effects of architectural or topographical factors on the gene and protein expressions of mesenchymal stem/stromal cells, the tendency of the cells to have a specific phenotype and on the cell proliferation (Zhang et al., 2016; Olvera et al., 2017; Barceló et al., 2023). One has to keep in mind that the cells used in this study were from menisci with a degeneration grade of two to four according to the Pauli classification (Pauli et al., 2011), without calcium deposition. It can be speculated that a proportion of the cells are already primed for osteogenic differentiation might be isolated and cultured, which could explain the higher expression of the osteogenic marker.

A recent study investigated the suitability of a scaffold made out of polyglycolic acid (PGA) fibers as a meniscus graft *in vitro* (Cojocaru et al., 2020). The human meniscal cells cultured on the scaffolds revealed a good viability/proliferation until day 7, with a slight decrease at day 14. This is in accordance with the cells showing an increase in cell viability when cultured on SPX for 7 days. Culturing the cells in 2D or on PGA with human serum or platelet concentrate resulted in the regulation of gene expression. A clear comparison with the present data is not possible due to the different conditions, but it displays the effects of culturing conditions and scaffolds on the cells.

For the *ex vivo* meniscus model the scaffolds were used in a tight-fit manner to ensure a tight contact between scaffold and donor meniscus. In the clinical situation of partial meniscus repair, the surgeon would also aim for a very close contact between the scaffold and the meniscal tissue to allow the invasion of the cells into the scaffold and therefore the regeneration procedure. The *ex vivo* meniscus model showed no ingrowth of cells into the scaffolds over a period of 7 days. The time frame might have been too short to let the cells migrate out of the meniscus matrix into the scaffold. The decision for the 7-day time point was made to reduce the risk of cell death in the *ex vivo* meniscal tissue and is a time point previously used in comparable experiments (Kang et al., 2006; Freymann et al., 2012; Zhang et al., 2016; Kwak et al., 2017; Ruprecht et al., 2019). In contrast, the 28-day cultivation of SPX in meniscal tissue resulted in ingrowth of MCs into SPX. The higher cell migration from the meniscus into the scaffolds from day 7 to day 28 is in accordance with previous observations using porcine menisci and porcine meniscus-derived matrix (Ruprecht et al., 2019). The lack of cell migration on day 7 might be explained by too weak or absent migration-stimulating signals during the 7 days and therefore the cells embedded in the dense extracellular matrix of the meniscus were not stimulated to grow out. However, the 28-day period was sufficient to stimulate cell ingrowth into the SPX. The analysis of gene expression showed that the expression of RUNX2, ALP, SOX9, and ACAN was increased in cells that migrated from meniscal tissue to SPX (both micro- and macro-porous) and CMI after 28 days of co-cultivation. This indicates that a greater number of cells were able to migrate into the scaffolds. However, it remains uncertain whether the cells differentiated into an osteogenic or chondrogenic lineage, and it appears that more time might be required for the cells to undergo this process.

## 5 Conclusion

In conclusion, this study investigated the potential of meniscal regeneration using graft materials by *in vitro* and *ex vivo* experiments. The results indicate that the use of SPX as a graft material is promising for meniscal tissue regeneration. Consistent with our findings, a case study indicated that the utilization of SPX block implants in a patient with bilateral medial meniscal lesions resulted in rapid integration of the implant, along with favorable radiographic and functional outcomes (Behrendt, 2023). Cells cultured on SPX showed increased viability and synthesis of the extracellular matrix protein collagen I compared with other materials. Consistent with this, the *ex vivo* organ culture demonstrated that meniscal cells exhibited migration from the surrounding tissue and successfully grew on the SPX scaffold within a 28-day period.

The study also highlights the importance of donor variation and the inhomogeneous structure of the meniscus, which may contribute to differences in gene expression and the cellular properties. The results suggest that meniscal cell phenotype and behavior may be influenced by factors such as cultivation conditions, scaffold architecture, and pore size.

Overall, the biocompatible materials show potential to promote cell growth, proliferation, and collagen production, which are critical for meniscal tissue regeneration. Further studies are required to optimize scaffold design, culturing conditions, and migration-stimulating signals to improve the efficacy of meniscal regeneration therapies.

## Data Availability

The original contributions presented in the study are included in the article/Supplementary Material, further inquiries can be directed to the corresponding author.
